# miR-128-3p reduced acute lung injury induced by sepsis via targeting PEL12

**DOI:** 10.1515/med-2021-0258

**Published:** 2021-08-05

**Authors:** Shinan Liu, Shuai Gao, Zhaoyu Yang, Peng Zhang

**Affiliations:** Department of Thoracic Surgery, China Tianjin Medical University General Hospital, Tianjin, China

**Keywords:** miR-128-3p, MPVECs, acute lung injury, sepsis, PEL12

## Abstract

**Objective:**

Acute lung injury (ALI) caused by sepsis is clinically a syndrome, which is featured by damage to the alveolar epithelium and endothelium. In this study, we employed mice models of cecal ligation and puncture (CLP) and primary mice pulmonary microvascular endothelial cells (MPVECs) *in vitro* to investigate the effect of miR-128-3p in ALI caused by sepsis.

**Methods:**

miR-128-3p agomir or randomized control were injected into adult male C57BL/6 mice 1 week before the CLP surgery. We used miR-128-3p agomir or scrambled control to transfect MPVECs and then employed lipopolysaccharide (LPS) stimulation on the cells. Pellino homolog 2 (PELI2) was predicted to be a direct target of miR-128-3p via luciferase reporter assay. MPVECs were cotransfected with lentiviral vector that expressed PELI2 (or empty vector) as well as miR-128-3p-mimics 1 day before LPS stimulation in rescue experiment. Transcriptional activity of caspase-3, cell apoptosis rate, and the expression levels of miR-128-3p, interleukin-1β (IL-1β), interleukin-6 (IL-6), and PELI2 were analyzed.

**Results:**

Compared with the sham group, the lung of mice in the CLP group showed pulmonary morphological abnormalities, and the expression of IL-6 and IL-1β, caspase-3 activity, and apoptosis rate were significantly upregulated in the CLP group. Inflammatory factor levels and apoptosis rate were also significantly induced by LPS stimulation on MPVECs. Upregulation of miR-128-3p effectively inhibited sepsis-induced ALI, apoptosis as well as inflammation. miR-128-3p also played a role in antiapoptosis and anti-inflammation in MPVECs with LPS treatment. PEL12 upregulation in MPVECs alleviated miR-128-3p-induced caspase-3 activity inhibition and pro-inflammatory factor production.

**Conclusions:**

miR-128-3p enabled to alleviate sepsis-induced ALI by inhibiting PEL12 expression, indicating a novel treatment strategy of miR-128-3p for sepsis-induced ALI.

## 1 Introduction

Sepsis is a life-threatening syndrome characterized by the overactive systemic inflammation reaction caused by the infection of bacteria, fungi, and viruses [1]. The patients with severe sepsis present with microthrombus formation and subsequent dysfunction of various organs [2]. Acute lung injury (ALI) is a clinical syndrome consisting of various acute respiratory failure in low O_2_, which has been one of the most common complications of serious sepsis [3]. Pathophysiological researches suggest that during the progression of ALI, injury of alveolar epithelial cells and endothelium results in damage to epithelial permeability, pulmonary edema as well as acute respiratory failure [4]. Although plenty of research in this field have been done and some results are obtained [5], no available medicine has been promoted as the common treatment of ALI [6].

Sepsis is a life-threatening disease caused by an overactive immune response to pathogen infection [7]. It has been one of the susceptible clinical factors related to the incidence rate of ALI. ALI is a serious syndrome made up of different disorders of acute hypoxemic respiratory failure [8]. During the process of ALI induced by sepsis, inflammation and up-regulation of apoptosis pathway lead to damage of alveolar epithelium and increase in epithelium permeability and edematous fluid in the alveolar cavity [9]. Increased plasma levels of pro-inflammatory cytokines such as tumor necrosis factor-α (TNF-α) and IL-6 have been reported to predict mortality in patients with ALI [10,11]. One study also showed that the increase in proapoptosis proteins such as Bcl2-associated X protein (Bax) induced extensive apoptosis and epithelium damage in alveolar epithelium in ALI [12]. These results demonstrated that regulation of inflammation and apoptosis pathway might provide new opportunities for the improvement of sepsis-induced ALI.

microRNA (miRNA) is one kind of noncoding ribonucleic acid with small molecular mass that serves key roles in different pathological progresses, which include dysfunction of different organs caused by sepsis [13]. *In vitro*, the overexpression of miR-146a alleviates myocardial dysfunction induced by sepsis through suppressing nuclear factor-κ-gene binding (NF-κB) activation as well as inflammatory cytokine expression [14]. miR-27A plays a role in liver protection of mice with sepsis induced by paclitaxel via inhibiting NF-κB/Tab3 signal pathway [15]. It has been verified that miR-128-3p plays a role in the progression of caner, neuropathic pain, and other diseases [16,17]. Cai et al. found that miR-128-3p induces cells with antiapoptotic effects and inhibits caspase-3 activation [18]. What’s more, miR-128-3p posttranscriptionally could inhibit WISP1 to suppress apoptosis and inflammation in human articular chondrocytes via the PI3K/AKT/NF-κB-signaling pathway [19]. Nevertheless, it has not been clear whether miR-128-3p has effects on sepsis-induced ALI.

In our research, we focused our attention to analyze the effects of miR-128-3p in ALI caused by sepsis and its potential molecular mechanism using mice sepsis models and primary mice pulmonary microvascular endothelium cells (MPVECs). The degree of lung tissue injury, cellular apoptosis, and inflammatory factor expression were tested. Our findings may provide new treatment of ALI caused by sepsis.

## 2 Materials and methods

### 2.1 Mice models with sepsis

A total of 24 adult male C57BL/6 mice ranging from 28 to 32 g were bought from Chinese Charles River Laboratories and were kept under a controlled environment (dark–light cycle with alternation in 12 h, at 22–24°C, and humidity of 60%) with accessibility of fodder and water. Our study experiments were accepted by Animal Care and Use Committee in Central Hospital in Wuhan City and were conducted according to the Care and Use of Laboratory Animals Guide [20]. Cecal ligation and puncture (CLP) operation was performed on 18 mice so as to build the model of sepsis resulting from CLP as mentioned above after 7 days of domestication [21,22]. In sum, mice were anesthetized by intraperitoneal injection of 10% chloralhydrate (3 mL/kg; Sigma Aldrich, St. Louis, Mississippi, USA) and were supinely performed on the operation tables. A longitudinal midline incision of 4 mm helped to expose the cecum. The cecum exposed to air was ligated by 10 mm from the end via 3-0 sutures, followed by the puncture with a 20-gauge needle at 5 mm distal from the ligation. The bowels were relocated after extruding little feces through gentle squeezing of the cecum and sterile sutures were utilized to close the abdominal muscle, skin, and peritoneum. We injected 5 mL/100 g saline to achieve fluid resuscitation sc. after operation. Same surgeries (*n* = 6) were performed on mice in sham group without ligation or cecum puncture.

### 2.2 Building models with low expression miR-128-3p

CLP mice were randomly assigned to three groups: CLP group, CLP plus native control (NC) group, and CLP plus miR-128-3P group. miR-128-3p agomir, miRNA reagents, and the control (NC agomir) were bought from Shanghai Genepharm Company, which were combined with linear polyethylenimine nano molecules (Sigma Aldrich), as previously mentioned [23]. One week before CLP, 200 μL mixture with 5 nmol miRNA (miR-128-3p or NC agomir) were injected into the veins in mice tails. Carbon dioxide asphyxia was used to euthanize mice 24 h after operation. The lungs were harvested and were properly maintained for further analysis.

### 2.3 Histopathological examination (HE) staining

HE was performed on the same parts of lung samples in mice. Paraformaldehyde of 4% was employed to maintain tissues that were kept in paraffin. After that they were cut into 5 μm slices. Hematoxylin and eosin (H & E) was used to stain these sections. Five fields were randomly selected and then each slide was graded under magnification of 400×.

### 2.4 Cellular culture and transfection

Mimics of control and miR-128-3p were synthesized through QUAGEN. HiPerFect transfection reagents (Qiagen, Valencia, CA, USA) were utilized to transfect miRNAs according to the manufacturer’s instructions while liposome 2000 was used to transfect other genes.

### 2.5 Assay of luciferase reporter

Peli2 3′-untranslating region fragments possessing target sites of mutant-type or wild-type miR-128-3p were cloned in luciferase gene in the pmirGlo vector (pmiR-Report). Renilla luciferase carrier (PRL-SV40) was used as normalized contrast. Plasmid of mimics of miR-128-3p and pmiR-Report was co-transfected with BALF microphage. According to instructions from manufacturers, activities of luciferase were detected 42 days later via assay of luciferase reporter (Promega, Madison, WI, USA) after transfection.

### 2.6 Quantitative real-time PCR (RT-qPCR)

mirVana miRNA isolation kit (Ambion) was utilized to collect total RNA including miRNA. mRNA of 1 μg was reversely transcribed to circular DNA through high-capacity circular DNA reverse transcription kit. The miRNA first-strand circular DNA was synthesized via miSCript II RT kit from Qiagen company. The gene expression was analyzed by SYBR Green kit from Sigma Aldrich. Primers were listed as follows:

miR-128-3p upstream: CTGGTAGGTCACAGTGAACCG,

downstream: TCAACTGGTGTCGTGGAGTC;

U6 upstream: TCGCTTCGGCAGCACATATAC,

downstream: CGCTTCACGAATTTGCGTG.

miR-128-3p expression was normalized through U6.

### 2.7 Enzyme-linked immunosorbent assay (ELISA)

Fluid of bronchoalveolar lavage from different rat groups was collected so as to measure the secretion of TNF-α using TNF-α ELISA kit from Abcam Company according to manufacturer’s instruction. The expression of pellino homolog 2 (PELI2) protein in homogenate lung tissue was examined by ELISA kit designed by Cusabio Biotech.

### 2.8 Cellular activity examination via caspase-3

Caspase-3 expression in lungs of mice and MPVECs was evaluated through caspase-3 Assay (Abcam, Cambridge, UK) following protocols given by the manufacturer. We homogenized lung samples in RIPA buffer with protease inhibitor. Our team lysed MPVECs within lysis of cells buffer half an hour after lipopolysaccharide (LPS) treatment. Protein concentration of all lung samples was modulated from 100 to 150 µg protein per 50 µL buffer of cell solution. The stuff buffer in 50 μL 2× protein lysate as well as 10 mM dithiothreitol were added to 50 μL samples of lungs, which was incubated in 5 μL substrate of 4 mM DEVD-p-NA under 37°C for 120 min. Microplate reader was employed to measure the optical density value of peroxidase reaction product at 450 nm.

### 2.9 Cell apoptosis detection via TUNEL

Apoptosis in lung tissues was understood through terminal deoxynucleotidyl transferase 2′-deoxyuridine 5′-triphosphate nick end labeling (TUNEL) staining kit (Roche Diagnostics). Apoptosis rate was determined by the ratio of the number of TUNEL-positive cells staining with DAPI in total cell numbers.

### 2.10 Western blot

Radioimmunoprecipitation assay (RIPA) plus inhibitor of protease cocktail was used to extract cells. Protein lysate of 40 μg was lysed in 12% sodium dodecyl sulfate–polyacrylamide gel and was later transferred to cellulose nitrate membrane. After 1 h block within 5% nonfat dry milk, these membranes were incubated with the primary antibodies (1:1,000) under 4°C for one night and was washed the second day. One hour after reaction with poly(butylene succinate-co-terephthalate) (PBST), these membranes were incubated with the secondary antibodies (1:5,000) under room temperature. Target proteins were examined via chemiluminescence assay kits from SuperSignal West Pico PLUS. PELI2 antibodies were purchased from Abcam Company (Shanghai, China).

### 2.11 Statistical analysis

Data are presented as the mean ± standard deviation (SD). Two groups’ differences were utilized through unpaired *t* tests. The differences between three or more groups were evaluated using one-way ANOVA. All the above analyses were performed by SPSS. *p* < 0.05 was considered statistically significant.

## 3 Results

### 3.1 miR-128-3p was downregulated in sepsis mice resulting from CLP

**Figure 1:**
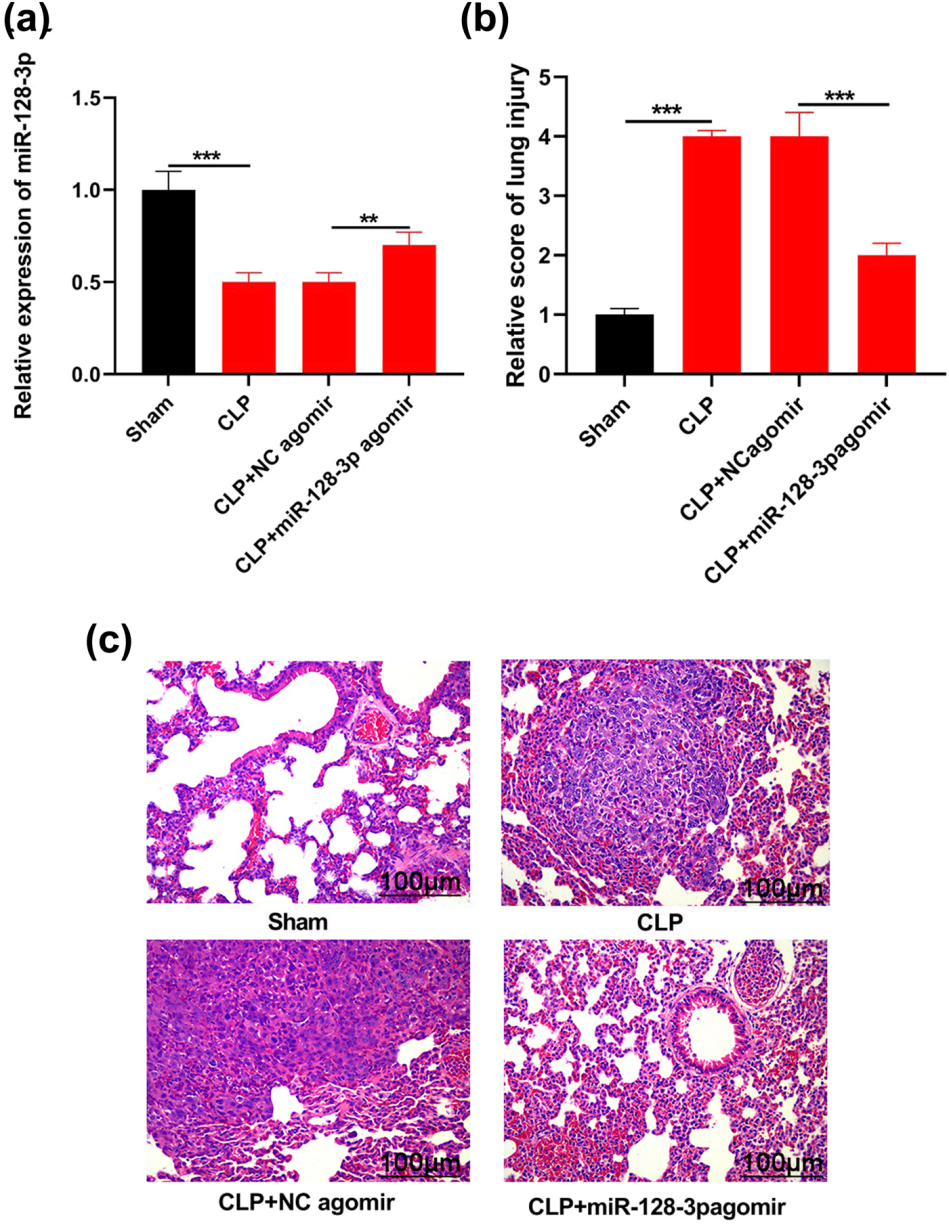
miR-128-3p expression on lung of mice models of sepsis due to CLP and miR-128-3p over-expression to lung damage as a result of sepsis. (a) RT-qPCR detected miR-128-3p level. (b) Measured score of lung damage on tissues with high miR-128-3p expression. (c) H & E staining tested the degree of lung damage in tissues with high miR-128-3p expression. **P < 0.01, ***P < 0.001.

In this study, a mouse septicemia model induced by CLP was established to study the regulatory effect of miR-128-3p on sepsis-induced ALI. Intravenously injecting miR-128-3p before CLP treatment led to induced miR-128-3p expression in sepsis mice. miR-128-3p expression in lung tissue prominently decreased after CLP operation when compared with sham group. Compared with those administering control miRNA, injection of plasmid, which expressed miR-128-3p, obviously enhanced miR-128-3p expression (Figure 1a; *P* < 0.05). Lung injury scoring was used to assess the degree of lung tissue damage, in which miR-128-3p overexpression significantly enhanced histological damage resulting from CLP surgery (Figure 1b). Normal structure of alveoli in sham group of mice was demonstrated in lung sections stained with H & E. In contrast, thickened alveolar septa and walls as well as collapsing alveolar sacs were shown in mice with CLP. miR-128-3p induction could reduce damage to alveoli caused by sepsis (Figure 1c), suggesting that miR-128-3p played a protective role in the lung damage induced by sepsis after CLP operation.

### 3.2 miR-128-3p induction reduced apoptosis indicator activity and pro-inflammatory factor production in the lung tissues of mice with CLP

**Figure 2:**
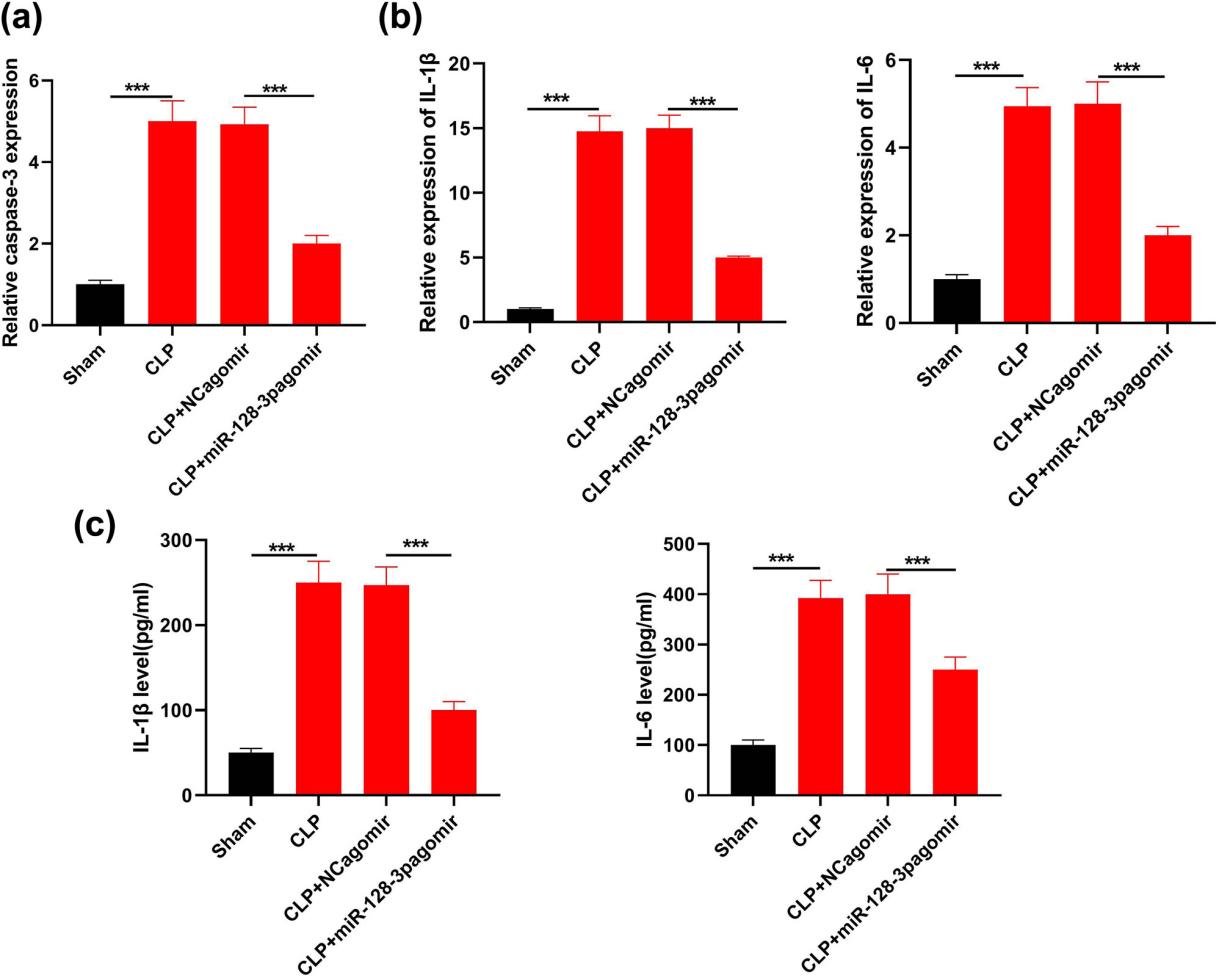
Effects of miR-128-3p overexpression on cellular apoptosis and inflammation among pulmonary cells of mice models of sepsis resulting from CLP. (a) Caspase-3 transcription activity in tissues with high miR-128-3p expression was detected via caspase-3 assay. (b) IL-6 and IL-1β mRNA levels were measured through qRT-PCR. (c) IL-1β and IL-6 were detected through ELISA. ***P < 0.001.

It was realized that caspase-3 transcription activity obviously increased in mice with CLP while miR-128-3p overexpression led to a great decrease in caspase-3 activity in these mice with sepsis (Figure 2a; *P* < 0.05). The mRNA and protein expressions of IL-1β and IL-6, two main pro-inflammatory factors, were both higher in mice with sepsis than in the sham group. miR-128-3p induction prominently suppressed IL-1β and IL-6 expression in mice with ALI (Figure 2b and c; *P* < 0.05). These data showed that miR-128-3p could improve cellular apoptosis induced by sepsis and pro-inflammatory cytokine production.

### 3.3 miR-128-3p overexpression could reduce apoptosis resulting from LPS as well as inflammation in MPVECs

**Figure 3:**
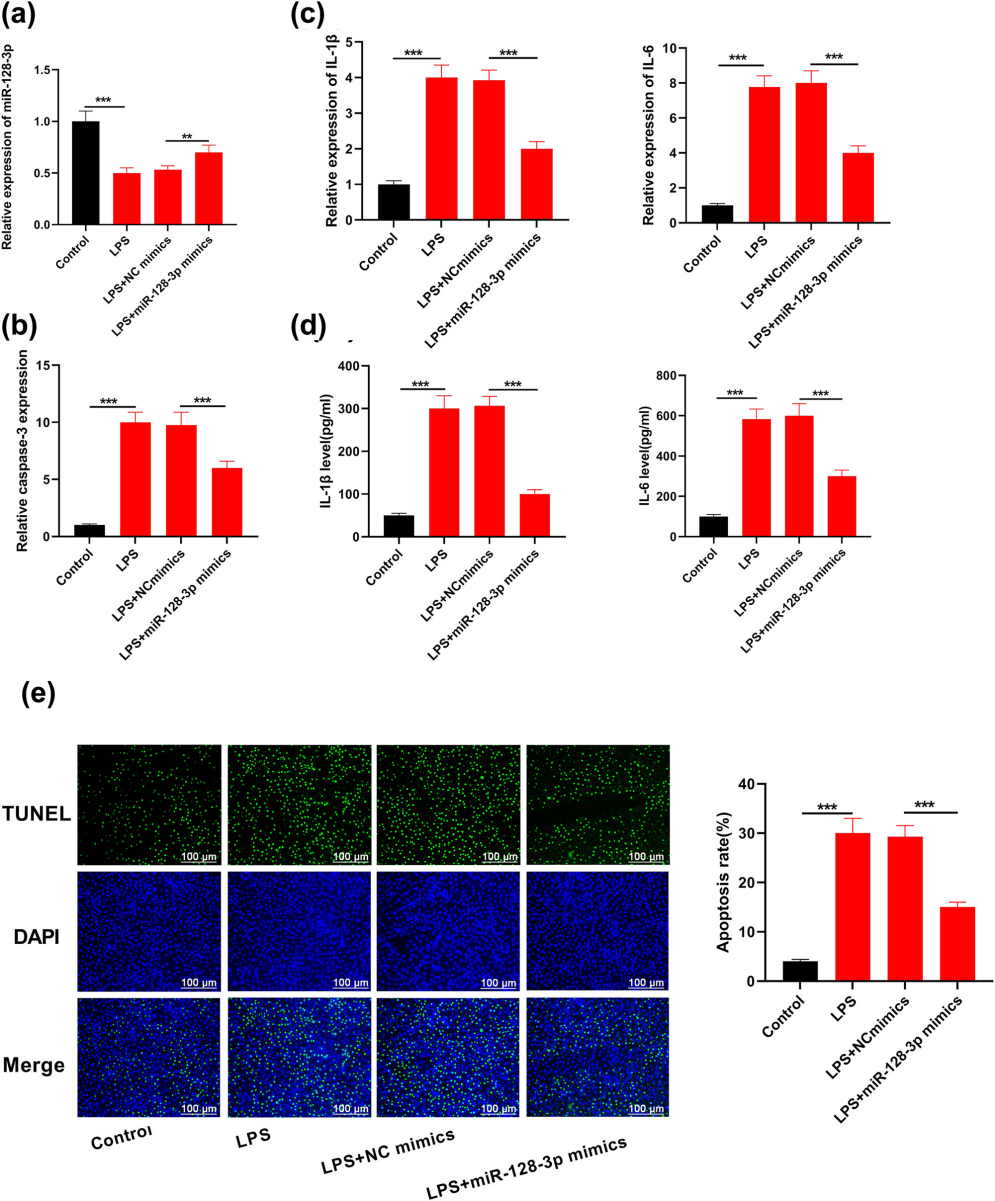
miR-128-3p influence on cellular apoptosis in MPVECs stimulated by LPS and inflammatory factors. (a) miR-128-3p level was tested in cells that were transfected with mimics of miR-128-3p using RT-qPCR. (b) Transcriptive activity of caspase-3 in cells transfected with miR-128-3p mimics was determined by caspase-3 assay. (c) IL-6 and IL-1β mRNA levels were measured through qRT-PCR. (d) TUNEL was utilized to determine apoptosis level in cells that were transfected using mimics of miR-128-3p. (e) TUNNEL assay evaluated cell apoptosis. ***P < 0.001, **P < 0.01.

In our study, lung injury was simulated via LPS stimulation of MPVECs. miR-128-3p expression prominently declined within cells with LPS treatment, compared with cells without LPS treatment. Under LPS stimulation, miR-128-3p levels were similar within both MPVECs of wild type and control mimics for transfection while the miR-128-3p transfection mimics prominently enhanced miR-128-3p expression (Figure 3a; *P* < 0.05). Caspase-3 activity prominently increased in MPVECs induced by LPS while miR-128-3p overexpression caused significant decrease in caspase-3 activity (Figure 3b; *P* < 0.05). In addition, miR-128-3p obviously inhibited IL-1β and IL-6 secretion from MPVECs induced by LPS (Figure 3c and d; *P* < 0.05). TUNEL showed that apoptosis of MPVECs was induced by LPS stimulation while miR-128-3p mimics transfection significantly decreased apoptosis rate compared to those transfected with control mimics (Figure 3e; *P* < 0.05). All the above consequences implied that miR-129-3p exerted the role of antiapoptosis *in vitro* and anti-inflammation.

### 3.4 PELI2 was the functional target by miR-128-3p

**Figure 4:**
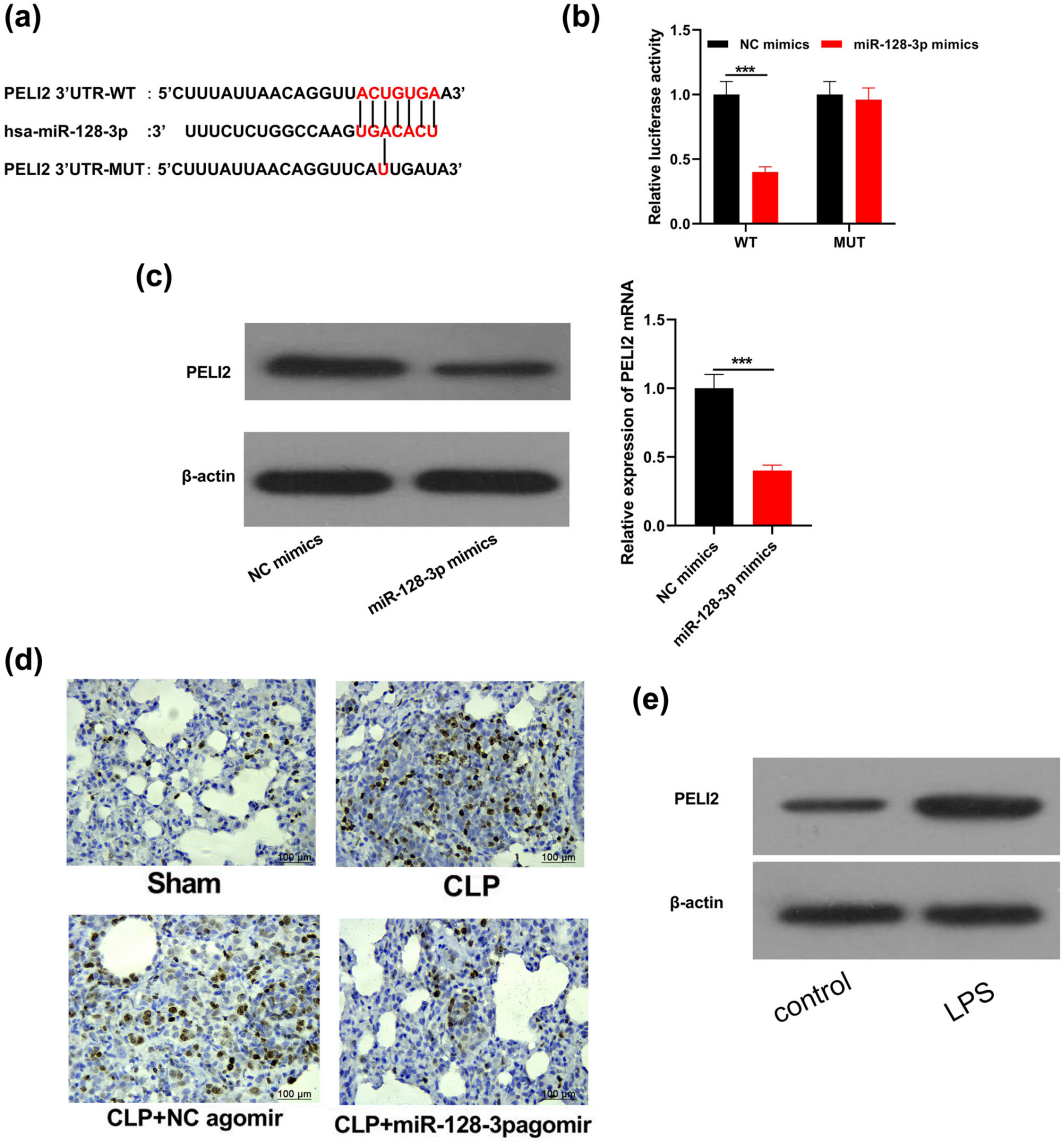
PELI2 is miR-128-3p target. (a) TargetScan data recognized that miR-128-3p contained conservative binding sites of PELI2. (b) The binding relation of PELI2 and miR-128-3P was determined via assay of luciferase reporter. (c) Western blot plus RT-qPCR analyzed PELI2 and its mRNA level in cells that were all transfected with mimics of miR-128-3p. (d) IHC of PELI2 expression in tissues. (e) Western blot analysis of PELI2. ***P < 0.001.

Next bioinformatics method, TargetScan, was employed to search for the downstream target of miR-128-3p for exploring the possible mechanisms of miR-128-3p in ALI. TargetScan is a widely used database that predicts biological targets of miRNAs by searching for the presence of conserved 8mer, 7mer, and 6mer sites that match the seed region of each miRNA [24,25]. The miRNA, which might regulate the overlaps as well as the miRNA-gene pairs, were screened with TargetScan [26]. PELI2 was forecasted to be direct miR-128-3P target gene accompanying a presumed combination site (Figure 4a). Luciferase reporter assay indicated that luciferase activity in PELI2-Wild-type cells transfected with mimics of miR-128-3p prominently declined compared with those using transfection to control mimics cells (Figure 4b; *P* < 0.05). However, no differences were observed in PELI2-MUT-type cells which were transfected with mimics of miR-128-3p or control mimic in terms of luciferase activity. Afterward we detected mRNA and proteins level of PELI2 in MPVECs wild types as well as those with overexpression of miR-128-3p and recognized that PELI2 expression obviously declined under the miR-128-3p induction (Figure 4c; *P* < 0.05). As shown in Figure 4d, the tissue levels of PELI2 in the different mice groups were also determined. PELI2 expression significantly enhanced due to the CLP surgery and alleviated miR-128-3p alleviated

PELI2 overexpression induced by CLP. PELI12 in the LPS-treated group were also increased *in vitro* (Figure 4e).

### 3.5 miR-128-3p suppressed cellular apoptosis and inflammatory reaction through PELI2 inhibition

**Figure 5:**
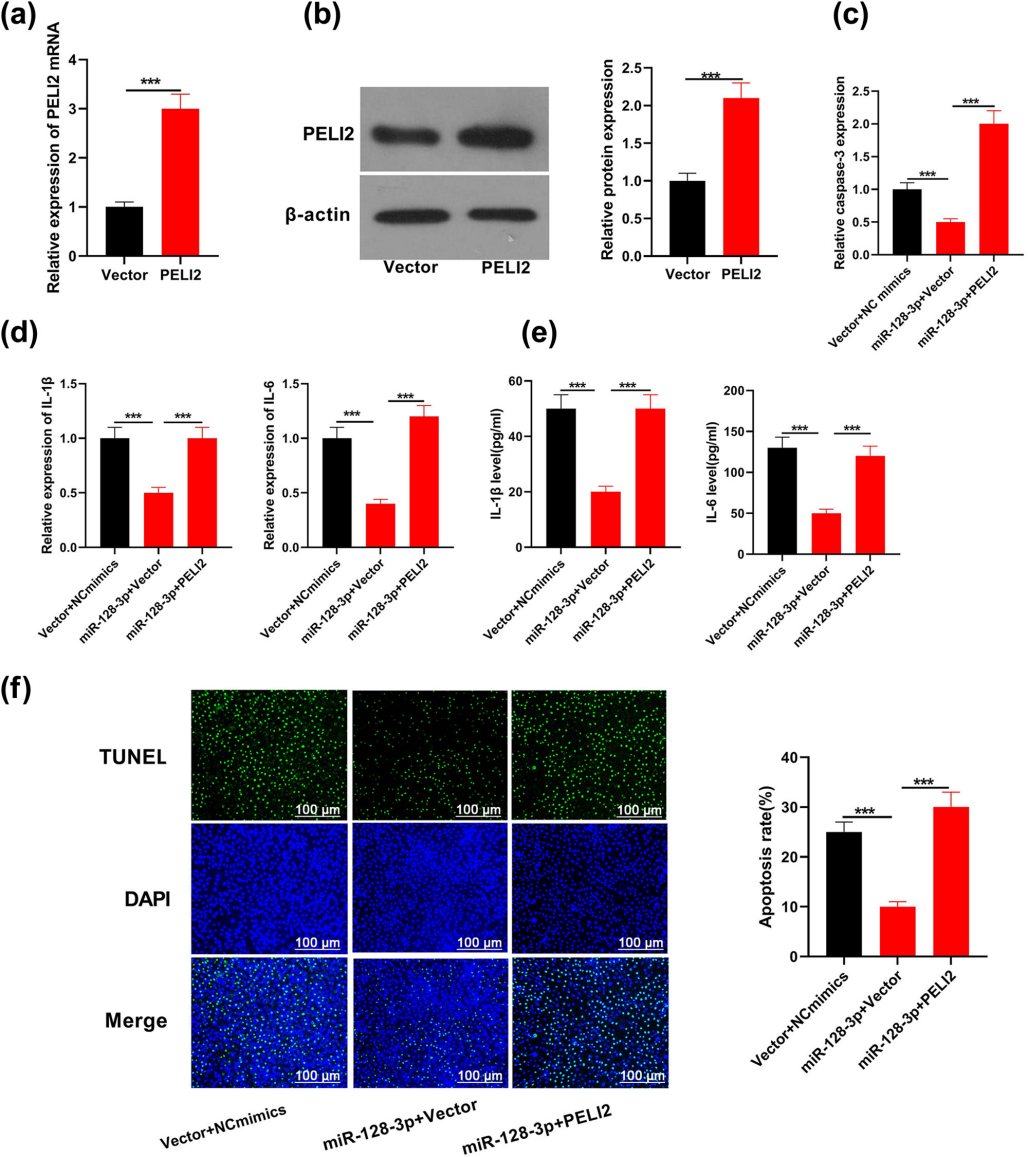
miR-128-3p took part in cellular apoptosis and inflammation via PELI2. (a and b) PELI2 and its mRNA level in cells with high expression of PELI2 were measured using RT-qPCR. (c) Transcriptive activity of caspase-3 in cells that were transfected both with mimics of miR-128-3p and plasmids overexpressing PELI2 was determined using caspase-3 assay kit. (d) RT-qPCR measured IL-6 and IL-1β mRNA level within cells which were transfected both with mimics of miR-128-3p and plasmids overexpressing PELI2. (e) IL-6 and IL-1β in supernatant of cells that were cotransfected with mimics of miR-128-3p in addition to plasmids overexpressing PELI2 was measured using ELISA. (f) TUNEL determined the apoptosis level in cells which were purely transfected with mimics of miR-128-3p and plasmids overexpressing PELI2. ***P < 0.001.

miR-128-3p mimics and lentiviral vectors (or empty vectors) expressing PELI2 were co-transfected into MPVECs to explore whether miR-128-3p regulated cell apoptosis as well as inflammation by targeting PELI2. PELI2 protein level significantly enhanced in MPVEC transfected with PELI2 vector, indicating transfection efficacy (Figure 5a and b; *P* < 0.05). We further discussed the effects on apoptosis and inflammation of MPVECs carrying miR-128-3p mimics brought by PELI2 overexpression. Compared with the control group, caspase-3 activity in MPVECs transfected with miR-128-3p as well as empty vectors greatly reduced. Nonetheless, PELI2-induced caspase-3 activity recovered to the level as in control group (Figure 5c; *P* < 0.05). miR-128-3P overexpression significantly inhibited the production of pro-inflammatory factors in MPVECs; however, both IL-1β and IL-6 prominently increased by induction of PELI2 in terms of mRNA and proteins level (Figure 5d and e; *P* < 0.05). Besides, miR-128-3p overexpression largely inhibited MPVECs apoptosis while transfection with vector expressing PELI2 significantly promoted apoptosis of MPVECs (Figure 5f; *P* < 0.05). In conclusion, these results implied that miR-128-3p inhibited apoptosis and inflammatory responses in ALI were caused by sepsis by target gene suppression, PELI2.

## 4 Discussion

Thirty years ago, sepsis caused in-hospital mortality to be over 80%, which was one urgent issue and it is still a life-threatening disease even under better wardship conditions and more advanced treatments, with mortality rate approaching 20–30% [27]. It has been verified that miRNAs exerted important roles in cell proliferation, apoptosis, inflammatory regulation, lymphocyte response, and regulation of sepsis [28]. In our research, it was reported for the first time that miR-128-3p was able to slow ALI progression resulting from sepsis *in vivo* as well as *in vitro*. In sepsis models, miR-128-3p overexpression decreased pro-inflammatory factors IL-6 and IL-1β’s expression as well as inhibited apoptosis indicator caspase-3 transcriptive activity. Further studies showed that miR-128-3p decreased apoptosis as well as inflammatory response in ALI induced by sepsis through negative modification of downstream target PELI2. PELI2, a positive regulator in LPS/TLR4 pathway, has been suggested to play a role in IL-1/LPS-induced activation of ERK and JNK MAPK pathways and increased stabilization of mRNAs encoding pro-inflammatory proteins [29,30]. You et al. found that PELI2 was upregulated in acute respiratory distress syndrome model, and miR-802 carried a protective role against LPS-induced ALI by downregulating PELI2 [31].

Mice CLP model has similar pathophysiological characteristics to sepsis caused by human abdominal perforation and has been widely applied in the study of organ dysfunction caused by sepsis [32]. In our research, the sepsis-induced ALI mouse model was successfully established. Compared with the sham operation group, the septal alveolar walls of CLP group mice were thickened and the alveolar sacs collapsed. More cellular apoptosis and pro-inflammatory factor induction were the key to pathogenesis of ALI during sepsis [33]. IL-6 and IL-1β were two pro-inflammatory factors that were positively secreted during inflammatory cascade of ALI [34,35]. Suppressing apoptosis signals and inflammation might potentially improve lung damage resulting from sepsis. Caspase-3 is one important index for cell apoptosis. It has been reported that activity of caspase-3 increased in ALI animal models [36,37]. In our research, increase in caspase-3 activity, IL-6 and IL-1β production as well as cell apoptosis rate have been observed.

It was shown for the first time that miRNAs potentially played a role in ALI via mice models of lung injury resulting from LPS [38]. Further studies on the effects of miRNAs on ALI indicated that miRNAs might regulate inflammation and apoptosis pathway of ALI through targeting special molecules or modulating downstream genes [39]. It has been reported that downregulation of miR-155 inhibited cell apoptosis as well as inflammation in mice pulmonary cells caused by CLP via targeting SIRT1, enhancing survival rate [40]. miR-1246 loss reduced cellular apoptosis in mice with ALI, IL-1β release as well as infiltration of neutrophils through suppressing its downstream target ACE2 [41]. Some studies have suggested that miR-127 expression significantly decreased in lung damage *in vivo*; however, miR-127 probe could alleviate lung inflammation by regulating CD46 in macrophages [42]. In another animal research, upregulation of miR-146a inhibited IL-6, IL-1β, and TNF-α secretion in ALI model induced by LPS via suppressing TRAF-6 and IRAK-1 [43]. In our research, it has been considered that miR-128-3p level largely declined in mice when compared with that of sham group. miR-128-3p mimics given before CLP operation effectively alleviated lung damage caused by sepsis, decreased activity of caspase-3 and cell apoptosis, and inhibited induction of IL-1β and IL-6. miR-128-3p exerted anti-inflammatory as well as antiapoptotic effects in MPVECs after LPS.

Previous studies have verified that PELI2 plays a role in lung injury caused by sepsis [44], which mechanism might be related to PELI2 activation. In our study, PELI2 was predicted to be a direct downstream target of Mir-128-3P, which is associated with apoptosis and oxidative stress. miR-128-3p significantly reversed cellular apoptosis as well as inflammation related with ALI via enhancing the expression of PELI2 in MPVECs, which implied that miR-128-3p improved ALI caused by sepsis by suppressing its downstream target PELI2. In conclusion, we found that miR-128-3p exerted effects in protection of sepsis-induced ALI, and it could be achieved by targeting PELI2 with anti-inflammation and anti-injury. miR-128-3p potentially provides new molecular target for clinical therapy of ALI.
